# SC06, a novel small molecule compound, displays preclinical activity against multiple myeloma by disrupting the mTOR signaling pathway

**DOI:** 10.1038/srep12809

**Published:** 2015-09-02

**Authors:** Kunkun Han, Xin Xu, Zhuan Xu, Guodong Chen, Yuanying Zeng, Zubin Zhang, Biyin Cao, Yan Kong, Xiaowen Tang, Xinliang Mao

**Affiliations:** 1 Jiangsu Key Laboratory of Translational Research and Therapy for Neuro-psycho-diseases, Department of Pharmacology, College of Pharmaceutical Sciences, Soochow University, Suzhou, China; 2 Department of Neurology, The First Affiliated Hospital of Soochow University, Suzhou, China; 3 Department of Hematology, the First Affiliated Hospital of Soochow University, Suzhou, China; 4 Jiangsu Key Laboratory of Preventive and Translational Medicine for Geriatric Diseases, Soochow University, China; 5 Department of Oncology, Suzhou Municipal Hospital (East Campus), Suzhou, China

## Abstract

The mammalian target of rapamycin (mTOR) is extensively involved in multiple myeloma (MM) pathophysiology. In the present study, we reported a novel small molecule SC06 that induced MM cell apoptosis and delayed MM xenograft growth *in vivo*. Oral administration of SC06 to mice bearing human MM xenografts resulted in significant inhibition of tumor growth at doses that were well tolerated. Mechanistic studies revealed that SC06 selectively inhibited the mTOR signaling pathway but had no effects on other associated kinases, such as AKT, ERK, p38, c-Src and JNK. Further studies showed that SC06-decreased mTOR activation was associated with the downregulation of Raptor, a key component of the mTORC1 complex. SC06 also suppressed the phosphorylation of 4E-BP1 and P70S6K, two typical substrates in the mTORC1 signaling pathway. Notably, expression of Raptor, phosphorylation of mTOR and phosphorylated 4E-BP1 was also decreased in the tumor tissues from SC06-treated mice, which was consistent with the cellular studies. Therefore, given the potency and low toxicity, SC06 could be developed as a potential anti-MM drug candidate by disrupting the mTOR signaling.

The mammalian target of rapamycin (mTOR) is a serine/threonine kinase that belongs to the phosphatidylinositol 3-kinase-related kinase (PIKK) superfamily including mTOR, ATM, ATR, hSMG-1, and DNA-PK. These proteins play an essential role in maintaining DNA stability, protein translation and glycogen synthesis^1^. In particular, mTOR coordinates cell growth and proliferation in response to inputs of growth factors, nutrient status and energy stress, thus regulating cell cycle progress and survival^1^. In mammalian cells, mTOR exists and acts as the catalytic subunit in two distinct complexes, mTOR complex 1 (mTORC1) and mTORC2, and regulates different biological functions. The major components in mTORC1 include mTOR, regulatory-associated protein of mTOR (Raptor), mammalian lethal with SEC13 protein 8 (MLST8), PRAS40, and DEPTOR. The major difference in mTORC2 is that mTORC2 contains rapamycin-insensitive companion of mTOR (Rictor) instead of Raptor in mTORC1. Therefore, Raptor and Rictor determine the assembly, localization and substrate binding of mTORC1 and mTORC2, respectively^2^. In complexing with other proteins, mTORC1 phosphorylates 70 kDa ribosomal protein S6 kinase I (P70S6K) and factor 4E-binding protein 1 (4E-BP1) translational repressor, thus modulating ribosome biogenesis and the translation of proteins that promote cell growth and division in response to intra-cellular as well as extra-cellular stimuli, such as stress, nutrients, energy, oxygen, and growth factors^2^,^3^,^4^. Although it is not well known the detailed function of mTORC2, its activation leads to phosphorylation of AKT and SGK1 (serum and glucocorticoid-regulated kinase 1), thus probably also being involved in cell proliferation and survival^5^.

By transducing both internal and external cellular signals, mTOR functions as a sensor of nutrients and energy, thus controlling protein synthesis and a broad panel of cell biological activities in both normal and cancer cells^3^. In cancer cells, paracrine or endocrine growth factors stimulate mTORC1 activity thus ensuring rapid growth and high proliferation^3^. As a catalytic subunit, the biological function of mTORC1 depends on the integrity of the complex. A potential anticancer drug clioquinol inhibits mTOR activity by disrupting the integrity of mTORC1 in multiple myeloma (MM)^6^, a fatal malignancy of plasma cells that leads to multiple organ dysfunction and universal death. MM is the second leading cause of death associated with blood cancers. It is well known that mTORC1 is a key modulator in MM cell proliferation, tumor development and chemoresistance, while disruption of this signaling could lead to MM cell apoptosis, tumor regression, and extended survival period of MM patients^6^. Therefore, inhibitors of this pathway are a promising treatment of MM patients. In the present study, we report a small molecule compound SC06 (Fig. 1a) that disrupts the mTOR signaling pathway and displays potent anti-MM activity *in vitro* and *in vivo*.

## Results

### SC06 decreases MM cell viability and activates apoptotic signaling

**Figure 1:**
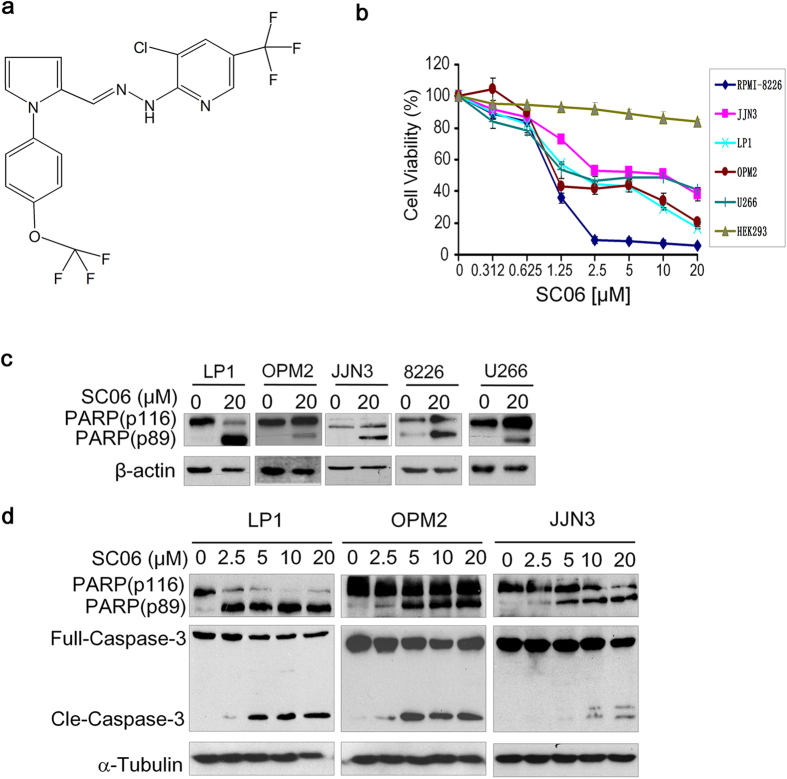
SC06 induces MM cell apoptosis. (**a**) the chemical structure of SC06. (**b**) SC06 inhibits MM cell viability. Five MM cell lines and HEK293 cells were treated with SC06 at indicated concentrations for 72 hr, followed by MTT assay. (**c**) MM cells (LP1, OPM2, JJN3, RPMI-8226, and U266 cells) were treated with 20 μM of SC06 or DMSO for 24 hr followed by the analysis of the expression of PARP and β-actin. (**d**) LP1, OPM2, and JJN3 cells were treated with SC06 at increased concentrations for 24 hr followed by the analysis of the expression of PARP, Caspase-3, and α-Tubulin.

**Figure 2:**
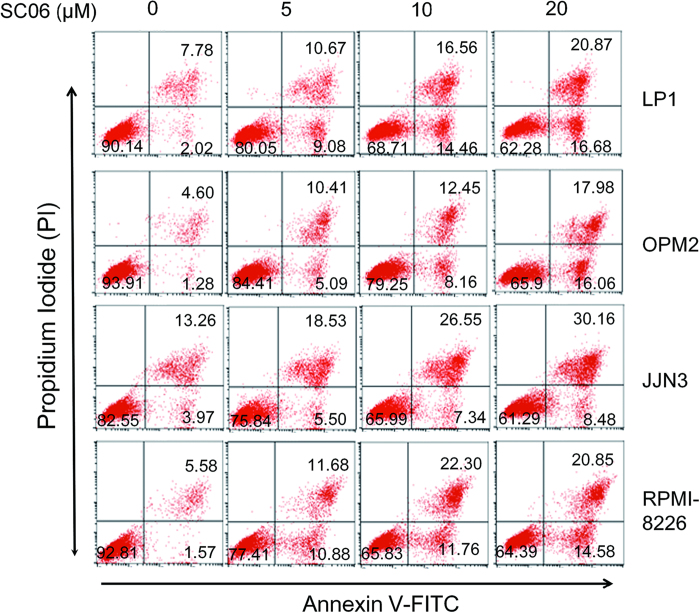
SC06 induces MM cell apoptosis. Myeloma cell lines LP1, OPM2, JJN3, and RPMI-8226 cells were treated with SC06 (0, 5, 10, 20 μM) for 24 hr. Cells were then stained with Annexin V-FITC and propidium iodide (PI), followed by analysis on a flow cytometer. The number of each quadrant was presented in %.

To evaluate the effects of SC06 on MM cell viability, a panel of MM cell lines and a non-MM cell line, HEK293 from the human embryonic kidney cells, were treated with increased concentrations of SC06 for 72 hr, followed by MTT assay as described previously^7^. As shown in Fig. 1b, SC06 decreased MM cell viability in a concentration-dependent manner. In contrast, SC06 had no marked effects on HEK293 cell viability. To find out whether cell apoptosis was also triggered, these MM cell lines were incubated with SC06 for 24 hr followed by immunoblotting assay to measure the apoptotic signaling. As shown in Fig. 1c, SC06 induced cleavage of PARP, a signature of cell apoptosis, in all examined cell lines. To further demonstrate this, LP1, OPM2 and JJN3 cells were incubated with increased concentrations of SC06 for 24 hr. Subsequent analysis revealed that both cleaved forms from PARP and Caspase-3 were increased in a concentration-dependent manner (Fig. 1d), suggesting the apoptotic signaling pathway was activated by SC06. To further demonstrated MM cell apoptosis induced by SC06, four MM cell lines were treated with SC06 for 24 hr at 0, 5, 10 or 20 μM, followed by Annexin-V-FITC and PI staining. Flow cytometric analyses demonstrated that SC06 increased Annexin-V positive cells (Fig. 2), which was consistent with the apoptosis analysis by immunoblotting (Fig. 1), suggesting that SC06 decreased MM cell viability and induced MM cell apoptosis.

### SC06 down-regulates the mTORC1 signaling pathway

**Figure 3:**
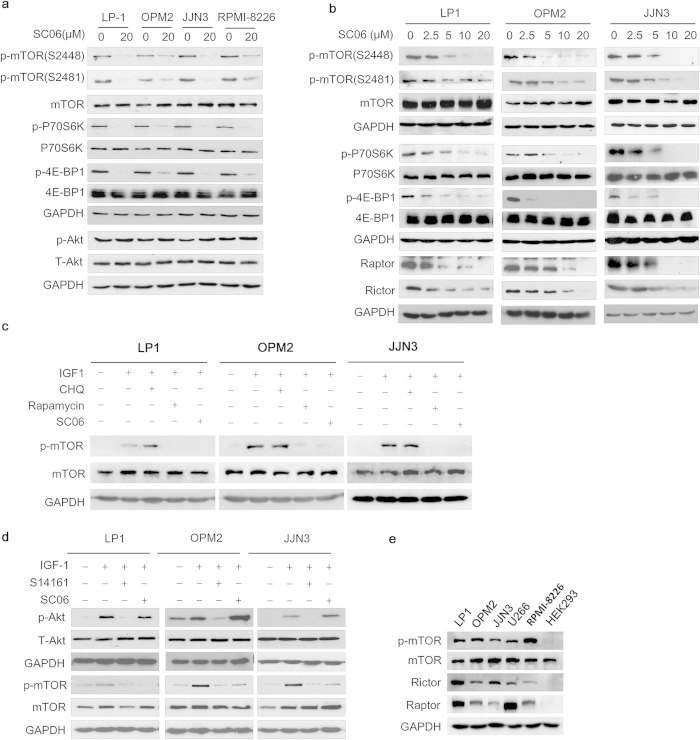
SC06 inhibits mTORC1 signaling pathway in MM cells. (**a**) MM cell lines (LP1, OPM2, JJN3, RPMI-8226) were treated with 20 μM of SC06 or vehicle for 24 hr. After incubation, cells were harvested and whole lysates were prepared for immunoblotting assay against indicated specific antibodies. (**b**) LP1, OPM2, and JJN3 cells were treated with SC06 at increased concentrations for 24 hr, followed by immunoblotting for specific proteins as indicated. (**c**) MM cells were starved overnight followed by SC06 (10 μM) or rapamycin (10 nM) or chloroquine (CHQ, 20 μM) for 2 hr, followed by IGF-1 (100 ng/mL) treatment for 15 min. Cell lysates were then prepared for immunoblotting assay against p-mTOR, mTOR, or GAPDH. (**d**) Starved MM cells were treated with SC06 or S14161 (10 μM) for 2 hr, followed by IGF-1 (100 ng/mL) treatment for 15 min. Cell lysates were then prepared for immunoblotting assay against indicated proteins. (**e**) Immunoblotting assay for p-mTOR at Ser2448, mTOR, Raptor, Rictor and GAPDH in MM cell lines (LP1, OPM2, JJN3, U266, RPMI-8226). HEK293 cells were used as a negative control.

Next we investigated the underlying mechanisms of SC06 in its anti-MM action. Because kinases are key targets of many anti-cancer drugs, we performed an immunoblotting assay to measure the effects of SC06 on various kinases. It turned out that SC06 potently suppressed the activation of mTOR in terms of phosphorylation in MM cells. As shown in Fig. 3a, SC06 suppressed mTOR phosphorylation at both Ser2448 and Ser2481 sites in all examined MM cell lines, along with decreased phosphorylation of P70S6K and 4E-BP1, two hallmark substrates of mTORC1. However, SC06 did not decrease the activation level of AKT, a major upstream modulator kinase of mTOR, or other mTOR-associated kinases such as ERK, p-38, c-Src, or JNK^10^,^11^,^12^,^13^ (Supplemental Fig. 1). Further studies demonstrated that SC06 inhibited mTOR and its downstream signals P70S6K and 4E-BP1 in a concentration-dependent manner (Fig. 3b). The effects of SC06 on mTOR were further confirmed in starved MM cells stimulated by IGF-1. IGF-1 significantly stimulated mTOR phosphorylation in 15 min, which was completely abolished by rapamycin, a specific inhibitor of mTOR. Similar to rapamycin, SC06 also attenuated mTOR activation in the presence of IGF-1 (Fig. 3c). However, chloroquine (CHQ), a lysosomal inhibitor, failed to inhibit mTOR phosphorylation. To exclude the effects of this inhibition was due to the PI3K/AKT signaling pathway, we next performed an experiment in which MM cells were treated with S14161^8^, a proven PI3K/AKT inhibitor, or SC06. As shown in Fig. 3d, S14161 abolished activation of AKT in all cell lines. Consistent with this finding, mTOR activation was also abolished by SC06, but SC06 had no effects on AKT activation. These results thus suggested that SC06 inhibited mTOR independent of AKT activation. Because mTOR activation is modulated by major components in the mTOR complex, we next evaluated the expression levels of phosphorylated mTOR, mTOR, Rictor and Raptor in MM cells. The results showed that all these proteins were highly expressed in all MM cell lines examined. In contrast, although the non-MM cell line HEK293 derived from human embryonic kidney expressed a high level of mTOR, a low level of activated mTOR was only detected. This finding was probably due to a low expression of Raptor and or Rictor (Fig. 3e). This expression pattern was consistent with cell viability affected by SC06. As shown in Fig. 1b, HEK293 cells were resistant to SC06, which probably due to the lack of activated mTOR. Therefore these results suggested that SC06 inhibited mTOR signaling pathway.

### SC06 downregulates the associated components of mTOR complex

mTOR is the catalytic subunit of mTORC1 and mTORC2, and its function depends on the associated proteins. Our previous study found that anti-cancer drug clioquinol inhibits mTOR activity by disrupting the mTOR complex^9^. Because SC06 suppressed mTOR activation in cells but failed to inhibit mTOR catalytic activity *in vitro* (Suppl. Table 1), we wondered whether SC06 had any effects on the associated components of mTOR complex. Because mTORC1 and mTORC2 largely depend on the regulatory subunits Raptor and Rictor, respectively, we measured their protein levels in the presence of SC06. As shown in Fig. 3b, SC06 downregulated both Raptor and Rictor in a concentration-dependent manner in all cell lines tested, including LP1, JJN3 and OPM2. In particular, SC06 decreased the Raptor level at a concentration as low as 2.5 μM (in LP1 cells) within 24 hr. At 10 μM, very low levels of Raptor could be detected in all tested cells, suggesting SC06 downregulated the key component of the mTOR complex.

### SC06 induces Raptor degradation via the proteasomal pathway

**Figure 4:**
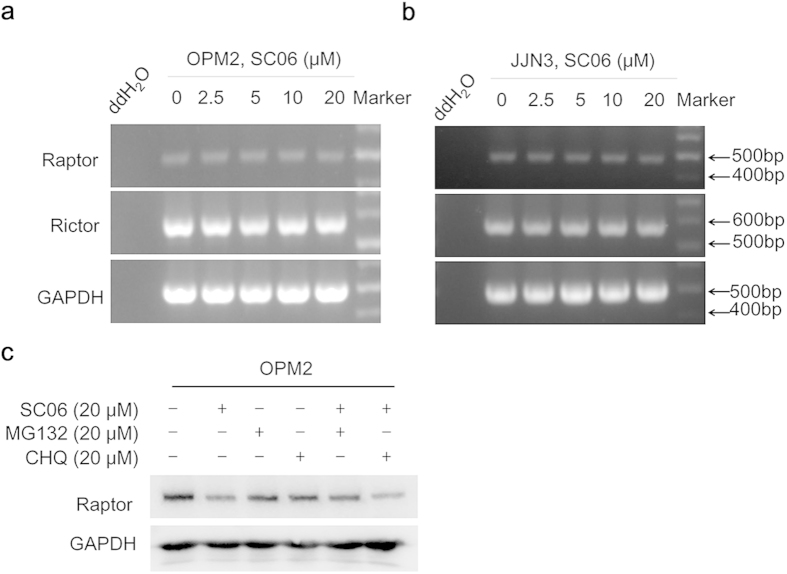
SC06 induces Raptor degradation via the proteasomal but not the lysosomal pathway. OPM2 (**a**) and JJN3 (**b**) cells were treated with SC06 at indicated concentrations for 24 hr followed by total RNA extract and reverse-transcription polymerase chain reaction (RT-PCR) for both Raptor and Rictor. (**c**) OPM2 cells were treated with SC06 alone or with MG132 or chloroquine (CHQ) for 8 hr. Cell lysates were then prepared for immunoblotting assay against Raptor. GAPDH was used as an internal control.

The above studies showed that SC06 could downregulate the expression of Raptor, a key scaffold protein in the mTORC1 complex. To find out the mechanism, we treated OPM2 and JJN3 cells with SC06, followed by RT-PCR to measure the transcription level of Raptor. As shown in Fig. 4a,b, SC06 had no effects on neither Raptor nor Rictor at the examined concentration range, but the same treatment significantly decreased the protein levels of both Raptor and Rictor (Fig. 3b). Protein stability is mainly modulated by lysosomes and proteasomes, therefore, to find out how SC06 downregulated these proteins, cells were treated with SC06 alone or together with MG132, a proteasomal inhibitor, or CHQ, a lysosomal inhibitor, followed by immunoblotting assay. As shown in Fig. 4c, MG132 but not CHQ prevented Raptor degradation, suggesting that SC06 induced Raptor degradation via the proteasomes. However, the detailed mechanism was yet to know.

### SC06 delayed MM tumor growth *in vivo* in association with disruption of mTOR signaling

**Figure 5:**
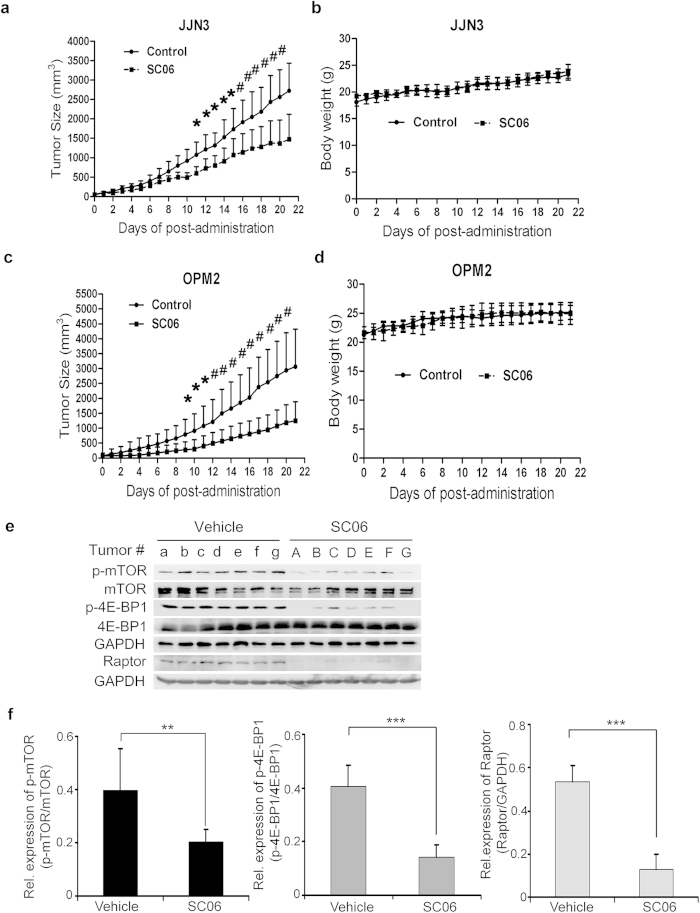
SC06 delayed MM tumor growth in association with disrupted mTOR signaling. MM cells JJN3 or OPM2 were injected subcutaneously into nude mice with a density of 30 million cells/site/mouse. When tumors were palpable, mice were randomly divided into two groups, one was orally given SC06 (50 mg/kg body weight) in PBS containing 10% Tween 80 and 10% DMSO daily for continuous 3 weeks. Another group was administrated with vehicle as a control. Tumor volumes and body weights were monitored every day. *p < 0.05; ^#^*p* < 0.01. (**a**,**c**) represented the tumor volume changes during the experimental course from JJN3 and OPM2, respectively. (**b**,**d**) represented the body weights of mice with xenograft from JJN3 and OPM2, respectively. (**e**) the expression of mTOR, 4E-BP1 and Raptor in the tumor species derived from JJN3 cells at the end of the experiments. GAPDH was used as a loading control. (**f**) Densitometry the relative expression levels of p-mTOR, p-4E-BP1 and Raptor based on signals in the immunoblots in e.

The above studies provided reliable evidence that SC06 decreased MM cell viability and induced MM cell apoptosis in association with disrupted mTOR signaling pathway. To evaluate its anti-myeloma efficacy *in vivo,* two independent human myeloma xenograft models were treated with SC06 by oral administration. As shown in Fig. 5, SC06 at 50 mg/kg/day led to a marked decrease in tumor growth in both MM models with JJN3 and OPM2 cell lines within 10 d (*p* < 0.05). At the end of the experiment of 3 weeks, SC06 decreased 42% and 52% of tumor volumes in the JJN3 and OPM2 models, respectively (Fig. 5a,c). But SC06 did not display marked effects on mice body weight in neither xenograft models (Fig. 5b,d).

To find out whether mTORC1 signaling was also decreased by SC06 in the xenograft model, JJN3-derived MM tumor tissue extracts were subjected to immunoblotting analyses. As shown in Fig. 5e, mTOR phosphorylation level was significantly inhibited in tumors from SC06-treated mice. This observation was supported by decreased 4E-BP1 phosphorylation and Raptor expression (Fig. 5e,f). Taken together, these results illustrated that SC06 delayed MM tumor growth *in vivo* in association with its disruption of the mTORC1 signaling pathway.

## Discussion

Dysregulated activation of mTOR signaling pathway is considered to be associated with drug resistance and poor prognosis of many cancers including MM^6^,^14^, ^15^,^16^,^17^. Last decade has witnessed mTOR as an anti-cancer target and many studies have demonstrated that inhibition of the mTOR signaling could be a promising strategy for MM therapy^6^,^18^. In the present study, we identified SC06, a novel small molecule, displays anti-MM activity by disrupting the mTOR signaling pathway. Although mTOR can be activated by the PI3K/AKT signaling and associated kinases, SC06 doesn’t affect the activation of these specific proteins including PI3K, AKT, ERK, p38, c-Src, and JNK.

Notably, SC06 does not show potent inhibition on mTOR activity in the purified enzymatic system, but it significantly inhibits mTOR activation in cells, suggesting that mTOR modulation by SC06 is probably due to its effects on the mTOR complex, e.g. disrupting the mTOR complex. As a catalytic subunit, mTOR exists in two complexes mTORC1 and mTORC2, in which the key component is Raptor and Rictor, respectively, which function as the specific scaffold proteins. Therefore, decreasing the expression of these two proteins could lead to reduced mTOR activity, because mTOR activity is dependent on the integrity of the complex. Impairment of any single components in the mTOR complex will reduce mTOR activity^3^. Previously, we found that a promising anti-cancer drug clioquinol inhibits mTOR activity via its action on the mTOR complex^9^. In the present study, SC06 does not modulate the expression of total mTOR, but downregulates the protein levels of Raptor and Rictor, suggesting SC06 probably disrupts both the integrity of mTOR complex, thus affecting their activity. We also found that SC06 has no effects on Raptor transcription but induces its degradation via the proteasomes although the detailed mechanism remains unclear. There are two dominant phosphorylation sites (Ser2448 and Ser2481) in mTOR. It is reported that the phosphorylation of Ser2448 was dependent on mTOR kinase activity and it is mediated by P70S6K because small interfering RNA-mediated P70S6K depletion reduces Ser2448 phosphorylation^19^. SC06 markedly suppresses the phosphorylation of mTOR at Ser2448, along with P70S6K, suggesting SC06 probably disrupts the phosphorylation feedback of mTORC1-P70S6K circuit. In addition, SC06 also decreases the phosphorylation of mTOR at Ser2481 that has been proposed as the site of mTOR-catalyzed autophosphorylation *in vitro* as mTOR intrinsic catalytic activity^20^. Rapamycin and amino acid withdrawal, although mediating the complete dephosphorylation of p70S6K, were reported to have no effect on the autophosphorylation of mTOR Ser2481 *in vivo*. In the present study, SC06 can inhibit mTOR phosphorylation at both Ser2448 and Ser2481, suggesting that SC06 can prevent both extrinsic and intrinsic catalytic activity of mTOR. This action also predicts that SC06 is a potent inhibitor of mTORC1 and a potent apoptosis inducer.

In agreement with the inhibition of mTOR signaling transduction, SC06 prevents MM cell viability and induces MM cell apoptosis. Notably, this finding is confirmed in tumors from mice treated with SC06. SC06 also decreases mTORC1 activation and its substrate 4E-BP1 phosphorylation as well as its scaffold Raptor in the tumors from mice receiving SC06, which further suggests that SC06 displays anti-MM activity in association with the suppression of mTORC1 pathway.

In summary, we identified a novel small molecule SC06 that showed potent inhibition on the activation of the mTORC1 signaling pathway in MM cells. Because of its potency *in vitro* and *in vivo*, SC06 could be developed as a promising anti-MM drug candidate.

## Methods

### Cell culture

MM cell lines LP1, OPM2, RPMI-8226, and U266 were purchased from American Type Culture Collection. JJN3, KMS11, OCI-MY5 and HEK293 were kindly provided by Dr. Aaron Schimmer from Ontario Cancer Institute, Toronto, Canada. All cell lines were maintained in Iscove’s modified Dulbecco’s medium (IMDM, Hyclone) supplemented with 10% fetal bovine serum, 100 μg/mL penicillin and 100 units/mL streptomycin (Beyotime Institute of Biotechnology, Nantong, China). Cells were cultured at 37 °C with 95% air and 5% CO_2_ in a humid incubator.

### Chemicals

SC06 or 3-chloro-2-(2-((1-(4-(trifluoromethoxy) phenyl)-1*H*-pyrrol-2-yl)methylene)hydrazinyc)-5-(trifluoromethyl)pyridine (Fig. 1A) was originally identified from our virtual screen^21^ and it was purchased from Thermo Fisher Scientific Inc (England, UK). Insulin-like growth factor-1 (IGF-1) was purchased from PeproTech (Rocky Hill, NJ, USA), Annexin V-FITC, propidium iodide (PI), 3-(4,5-dimethylthiazol-2-yl)-2,5-diphenyltetra sodium bromide (MTT), and dimethyl sulfoxide (DMSO) were purchased from Sigma (St Louis, MO, USA).

### Antibodies

Specific antibodies against PARP, Caspase 3, p-AKT(S473), AKT, p-mTOR(Ser2448), p-mTOR(Ser2481), mTOR, Raptor, Rictor, p-P70S6K, P70S6K, p-4E-BP1, 4E-BP1, ERK (p42/p44), p-ERK (p42/p44), p-p38, p-JNK, c-Src, and p-Src were purchased from Cell Signaling Technologies, Inc. GAPDH antibody was purchased from Abgent (Suzhou, China). Antibodies against β-actin and α-tubulin, as well as anti-mouse immunoglobulin G (IgG) and anti-rabbit IgG horseradish peroxidase conjugated antibody were purchased from R&D Systems.

### Cell viability assay

MM cells were plated at a density of 1 × 10^4^ cells per well into 96-well plates while HEK293 cells were plated at a density of 5000 per well one day before SC06 treatment. Cells were incubated for 72 hr with SC06 in a serial of concentrations from 0 to 20 μM. Cell viability was evaluated by MTT assay as described previously^22^.

### Flow cytometry

MM Cells (1 × 10^5^) were treated with increasing concentration of SC06 for 24 hr. Cells were then collected for staining with Annexin V-fluorescein isothiocyanate (Annexin V-FITC) and propidium iodide (PI, Sigma) for 5 min in dark. Stained cells were analyzed on a flow cytometer (FACSCalibur®, Becton Dickinson) as described previously^23^. In order to determine cell cycle, MM cells treated by SC06 were sequentially treated with 70% cold ethanol, DNase free RNase (Beyotime). Cell pellets were then stained with PI and analyzed on a flow as described previously^24^.

### mTOR activity assay *in vitro*

The inhibition assay of SC06 on recombinant mTOR was performed by Reaction Biology Corp., Malvern, PA. Detailed protocol was described previously^8^.

### Immunoblotting

Cells were first treated with SC06 at indicated concentrations for 24 hr. Then, cells were collected to prepare cell lysates for immunoblotting assay as described previously^23^.

### Phosphorylation analysis by immunoblotting

MM cells were starved overnight in Iscove’s modified Dulbecco’s medium that contained 0.5% fetal calf serum before treatment with SC06 or a PI3K inhibitor S14161 or an mTOR inhibitor rapamycin for 2 hr. Cells were then incubated with 100 ng/mL of IGF-1 for 15 min before being prepared in a lysis buffer designed for the analysis of phosphorylated proteins for 20 min as described previously^25^. After clarification at high speed at 4 °C for 30 min, equal amount (30 μg) of total proteins was subjected to SDS-PAGE separation and immunoblotting analysis with specific antibodies.

### Reverse Transcription-Polymerase Chain Reaction (RT-PCR)

MM cells were treated with SC06 (0, 2.5, 5, 10, 20 μM) for 24 hr. Total RNA was extracted using Trizol® Reagent (Invitrogen) according to the manufacturers’ instructions and quantitated using a Nanodrop spectrophotometer (Thermo Scientific). cDNA was synthesized from equal quantities of total RNA using the EasyScript First-Strand cDNA Synthesis SuperMix (TransGen Biotechnology). To determinate relative mRNA levels of Rictor and Raptor, the polymerase chain reaction was performed in a 25 μL reaction system containing 12.5 μL 2 × Taq Master Mix (Novoprotein), 2 μL of RT-reaction product, and 0.5 μM of each primer, and 9.5 μL ddH_2_O. The primers used were as follows: Raptor, forward 5′- ATTCTCGCCGTGATCGTCAA-3′ and reverse 5′- GGAGAAGGCAAGGCGTAGTT -3′; Rictor, forward 5′-AGAACCTCCGAGTACGAGGG-3′ and reverse 5′- GCCACCACCTCTGGATTCTG-3′. Reaction cycling conditions were 3 min at 95 °C, followed by 35 cycles at 95 °C for 30 s, 60 °C for 30 s, and 72 °C for 40 s, and 1 cycle at 72 °C for 10 min. Products were analyzed on 2% agarose gels.

### *In vivo* studies

Human MM cells (JJN3 or OPM2, 3 × 10^7^ cells/site) were injected subcutaneously in the right flanks of nude mice (5-6 weeks old, female, Shanghai Slac Laboratory Animal Co. Ltd., Shanghai). When tumors were palpable, mice in each model were randomly divided into two groups. One group was given SC06 (50 mg/kg body weight) in PBS containing 10% Tween 80 and 10% DMSO daily for 24 d, another group was given 10% DMSO as vehicle. Tumor sizes and mouse body weight were monitored over the duration as described previously^23^. To analyze protein signals from the tumor species at the end point of the experiment, tumors were excised and snap-frozen immediately in liquid nitrogen. Tissue samples were then minced and homogenized to extract whole cell lysates. The clarified supernatants of the samples were applied for immunoblotting analyses using specific antibodies. This *in vivo* study was conducted in accordance with the protocols approved by the Ethical Committee of Experimental Animals of Soochow University.

### Statistical Analysis

Data are presented as mean values with 95% confidence intervals (CIs) unless otherwise indicated. *t* test was used for comparisons of two groups in the *in vitro* studies. All statistical tests were two-sided, and a *p* value less than 0.05 was considered to be statistically significant.

## Additional Information

**How to cite this article**: Han, K. *et al.* SC06, a novel small molecule compound, displays preclinical activity against multiple myeloma by disrupting the mTOR signaling pathway. *Sci. Rep.* **5**, 12809; doi: 10.1038/srep12809 (2015).

## Supplementary Material

Supplementary Information
