# Supplementary material for: SC06, a novel small molecule compound, displays preclinical activity against multiple myeloma by disrupting the mTOR signaling pathway
Source: Sci Rep. 2015 Sep 2;5:12809. doi: 10.1038/srep12809 (PMC4556980; doi:10.1038/srep12809)
Supplement: Supplementary Information [file srep12809-s1.pdf]

SC06, a novel small molecule compound, displays preclinical activity against multiple myeloma by disrupting the mTOR signaling pathway

Kunkun Han[1], Xin Xu[1], Zhuan Xu[2], Guodong Chen[1], Yuanying Zeng[1], Zubin Zhang[1], Biyin Cao[1], Yan Kong[2], Xinliang Mao[1,3]

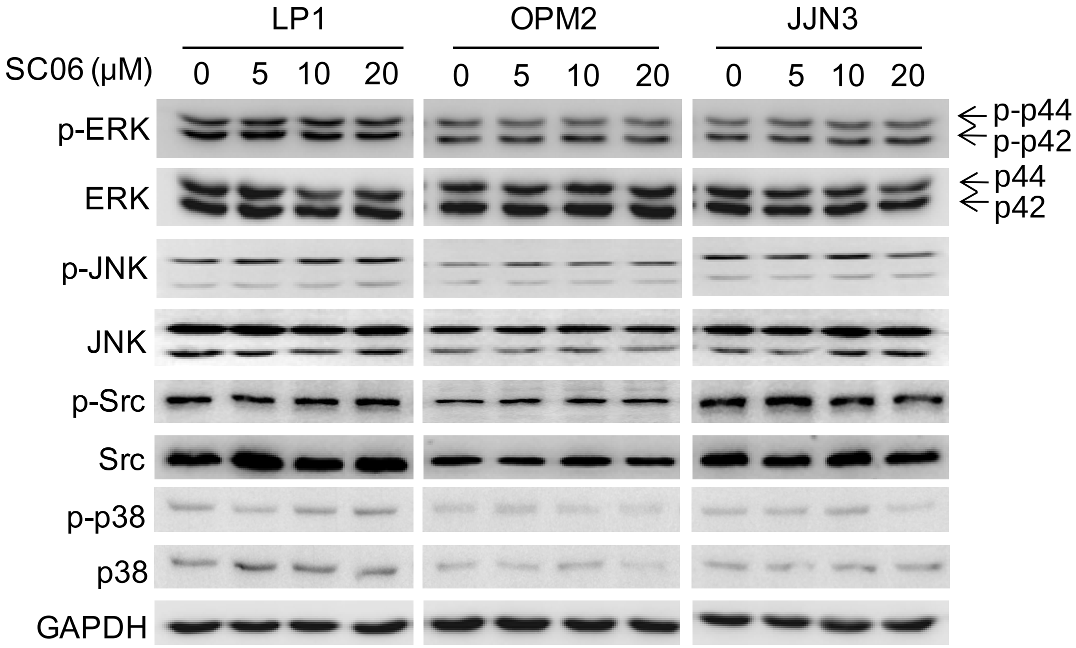

Supplemental Figure 1. SC06 does not affect mTORC1-associated kinases in MM cells. MM cell lines (LP1, OPM2, JJN3) were treated with SC06 at increased concentrations or vehicle for 24 hr. After incubation, cells were harvested and whole lysates were prepared for immunoblotting assay against with indicated specific antibodies.

Suppl. Table 1. SC06 doesn't inhibit mTOR activity in cell-free assay.

SC06 Conc.(M)	SC06 (%)	LY294002 (%)	LY294002 Conc.(M)
3.00E-04	96.25	26.36	2.00E-05
1.00E-04	96.70	49.01	6.67E-06
3.33E-05	100.55	73.49	2.22E-06
1.11E-05	100.36	94.86	7.41E-07
3.70E-06	102.84	99.40	2.47E-07
1.23E-06	102.14	104.86	8.23E-08
4.12E-07	101.75	102.34	2.74E-08
1.37E-07	100.06	97.71	9.14E-09
4.57E-08	101.34	106.11	3.05E-09
1.52E-08	95.87	96.08	1.02E-09
DMSO	101.68	100.00	DMSO
